# Microneedle-Mediated Permeation Enhancement of Chlorhexidine Digluconate: Mechanistic Insights Through Imaging Mass Spectrometry

**DOI:** 10.1007/s11095-022-03309-8

**Published:** 2022-06-10

**Authors:** Melissa Kirkby, Akmal Bin Sabri, David Scurr, Gary Moss

**Affiliations:** 1 School of Pharmacy and Bioengineering, Keele University, Keele, ST5 5BG UK; 2 School of Pharmacy, University of Nottingham, University Park, Nottingham, NG7 2RD UK; 3 School of Pharmacy, Medical Biology Centre, Queen’s University Belfast, 97 Lisburn Road, Belfast, BT9 7BL UK

**Keywords:** chlorhexedine, microneedles, skin Imaging, skin permeation, time of flight secondary ion mass spectrometry

## Abstract

**Purpose:**

Chlorhexidine digluconate (CHG) is a first-line antiseptic agent typically applied to the skin as a topical solution prior to surgery due to its efficacy and safety profile. However, the physiochemical properties of CHG limits its cutaneous permeation, preventing it from reaching potentially pathogenic bacteria residing within deeper skin layers. Thus, the utility of a solid oscillating microneedle system, Dermapen®, and a CHG-hydroxyethylcellulose (HEC) gel were investigated to improve the intradermal delivery of CHG.

**Methods:**

Permeation of CHG from the commercial product, Hibiscrub®, and HEC-CHG gels (containing 1% or 4% CHG w/w) was assessed in intact skin, or skin that had been pre-treated with microneedles of different array numbers, using an Franz diffusion cells and Time-of-Flight Secondary Ion Mass Spectrometry (ToF–SIMS).

**Results:**

Gels containing 1% and 4% CHG resulted in significantly increased depth permeation of CHG compared to Hibiscrub® (4% w/v CHG) when applied to microneedle pre-treated skin, with the effect being more significant with the higher array number. ToF–SIMS analysis indicated that the depth of dermal penetration achieved was sufficient to reach the skin strata that typically harbours pathogenic bacteria, which is currently inaccessible by Hibiscrub®, and showed potential lateral diffusion within the viable epidermis.

**Conclusions:**

This study indicates that HEC-CHG gels applied to microneedle pre-treated skin may be a viable strategy to improve the permeation CHG into the skin. Such enhanced intradermal delivery may be of significant clinical utility for improved skin antisepsis in those at risk of a skin or soft tissue infection following surgical intervention.

**Supplementary Information:**

The online version contains supplementary material available at 10.1007/s11095-022-03309-8.

## INTRODUCTION

Chlorhexidine digluconate (CHG) is a symmetric bis-biguanide molecule comprising two chloroguanide chains that are connected by a central hexamethylene chain. The molecule is typically applied as a topical solution to disinfect the skin prior to surgery. At physiological skin pH, this broad-spectrum biocide is positively charged and exerts its bactericidal effect by binding to the anionic phospholipids on the cell wall of cutaneous bacteria. This leads to the rupturing of the bacterial cell walls, leading to cytoplasm leakage and culminating in cell death (1). According to the National Institute for Health and Care Excellence (NICE) guidelines, CHG is the first line antiseptic for the prevention and treatment of surgical site infections (2). This is attributed to the ability of CHG to effectively and selectively eradicate cutaneous bacteria with minimal resistance and low mammalian toxicity (3). This is further corroborated by a systematic review and meta-analysis by Noorani *et al.* that showed that pre-operative skin disinfection with chlorhexidine is superior to other antiseptics, such as povidone–iodine, in mitigating postoperative surgical site infection after clean-contaminated surgery (4).

Despite the widespread and long term use of CHG in practice, the molecule exhibits poor skin permeability (5, 6) which is attributed to its high molecular weight (897.8 g/mol (7)) and low log P (0.0133) along with its propensity to be ionised at physiological skin pH (3, 8, 9)*.* The presence of the molecule in an ionised state on the skin surface is beneficial in enabling the molecule to exert its bactericidal effect but this also precludes the molecule from traversing the highly lipophilic *stratum corneum* and acting against deeper-lying bacterial pathogens within the skin. Some of the common bacteria that are associated with surgical site infection include *Staphylococcus aureus, coagulase- negative staphylococci, Enterococcus spp.* and *Escherichia coli* which in a majority of surgical site infections, originate from the patient’s endogenous dermal microbiome (10). Indeed, it is known that the dermal microbiome consist of bacteria that reside not only on the surface of the skin but also encompasses microbes that reside deep within the epidermis and dermis (11, 12). Some of the common bacteria that typically reside within the deeper strata of the skin include *S. epidermidis* and *Pseudomonas spp* (12)*.* In addition, it is estimated that these bacteria, also known as subepidermal bacteria, may reside well below the skin surface at a depth of approximately 400–700 μm (13). This group of bacteria harbours the opportunity to become pathogenic should the skin barrier become compromised, for example, at the site of a surgical incision.

Therefore, there is an impetus to improve the delivery of CHG deeper into the skin prior to surgical procedures in order to mitigate the likelihood of postoperative surgical site infection. Several strategies have been explored to improve the delivery of CHG into the skin, such as reformulating the antiseptic with poly(amidoamine) (PAMAM) dendrimers (14). One strategy yet to be explored in order to improve the delivery of CHG into the skin prior to surgical procedure is the use of microneedles. These biomedical devices are considered to be hybrids of the transdermal patch and the hypodermic needle which, upon application to the skin, generate microchannels within the *stratum corneum*. These channels can be utilised as conduits to improve the delivery of molecules into the skin that would otherwise suffer poor cutaneous permeation due to their inherent physiochemical properties (15). Such a delivery strategy would be ideal for the delivery of highly polar and water-soluble molecules such as CHG. This is because the microchannels generated are filled with interstitial fluid that could be utilised by ionised CHG molecules to diffuse into the skin without traversing the highly tortuous *stratum corneum* lipid permeation pathway. Several researchers have evaluated the utility of using microneedles, as a delivery strategy, to improve the delivery of antimicrobial compounds (antifungal and antibiotic) into the skin (16, 17). For instance, Peng *et al*. explored and demonstrated the utility of using dissolving microneedles to achieve localised and long-acting intradermal delivery of Amphotericin B, an antifungal agent, for the treatment of cutaneous fungal infections. In the work, the researchers demonstrated that the microneedles were able to be inserted into the skin to a depth of 300 µm resulting localised release of Amphotericin B into the dermis, the location where most fungal infections typically reside (18). Furthering this, Sabri *et al*. showed that the use of hydrogel-forming microneedles were capable of achieving high dose of antibiotic delivery into the skin. In the work, the researchers fabricated a hydrogel forming microneedle system from Gantrez® S-97 and Carbopol® 974P NF crosslinked with PEG 10,000 that was used in combination with an antibiotic loaded lyophalised wafer. The composite microneedle-based pharmaceutical system was capable of delivering 500 µg of cefazolin into the dermis within 2 h of application furthering highlighting the utility of microneedle as a delivery system for localised intradermal delivery of antimicrobial agents (19). Nevertheless, there is no publication to date that have demonstrated the capability of using microneedles as a potential physical permeation enhancer to promote the delivery of antiseptic agent such as CHG into the skin as a prophylaxis treatment against potential skin infection following surgical incision as most microneedle system developed has been focussed on the treatment of established skin infections.

In addition to improving the delivery of CHG into the skin, detailed analysis of the dermal distribution of the antiseptic within the skin strata is essential in order to elucidate and evaluate the permeation enhancement effect conferred by microneedles into deeper skin tissues. Conventionally, high-performance liquid chromatography (HPLC) is frequently utilised in microneedle permeation studies in order to evaluate the delivery of compounds into and across the skin. Although this method confers quantitative information pertaining to the permeation profile of the compounds of interest, the tissue manipulation steps and extraction procedures lead to the loss of valuable spatial information regarding drug localisation within the skin tissue (20). One additional, complementary technique that has been utilised in skin permeation research in tandem with HPLC analysis is time-of-flight secondary ion mass spectrometry (ToF–SIMS) (21). This analytical technique enables researchers to chemically map the compound of interest within a biological milieu via tracking the mass-to-charge ratio (*m/z*) of the specific molecular or fragment ions from the mass spectra (22). Furthermore, the capability of the instrument to track the topical permeation of a range of permeants, including immunomodulators (21, 23), ascorbic acid (20), dihydroquercetin (24), fatty acids (25), carvacrol (26) and roflumilast (27) highlights the versatility of the technique in skin research. Additionally, the secondary ion images generated enable researchers to visualise the homogeneity of drug distribution either laterally across the skin (via tape strip analysis) (14) in relation to depth of permeation (via skin cross-sectional analysis) (28). This imaging technique has previously been used to view native skin components (29, 30) in both healthy (31–33) and diseased tissue (34–36). Despite the versatility and capability offered by this technique, the application of ToF–SIMS in evaluating the effectiveness of microneedle-based drug delivery systems has been very limited and the application of such a technique to this field could potentially be of great value in expanding mechanistic insights into microneedle-based skin permeation.

CHG is an inherently poor permeant of the skin due to its physicochemical properties. It is also clear from the literature discussed above that a significant bacterial load may reside in areas of the skin that are no normally amenable to CHG permeation. Limited antisepsis is therefore delivered to these sites deeper in the skin tissue where a significant bacterial load may be found. As microneedles have been shown to improve depth permeation in the skin the assessment these technologies to improve skin deposition and therefore efficacy of CHG is a reasonable strategy which may offer improved outcomes. Thus, in the present work, we evaluated the utility of using a poke-and-patch strategy, using an oscillating solid microneedle system, Dermapen® with varying array numbers, to enhance the intradermal delivery CHG from hydroxyethyl cellulose (HEC) gel relative to Hibiscrub® (4% w/v CHG). Following a Franz-type diffusion cell study, the treated skin samples were analysed via HPLC in order to quantify the amount CHG that permeated into the *stratum corneum*, whilst ToF–SIMS analysis was implemented to visualise the distribution of the antiseptic within the skin.

## MATERIALS AND METHOD

### Materials

2-hydroxyethylcellulose (HEC, molecular weight ~ 1,300,000), trimethylamine (25% w/ v in water), HPLC grade methanol (> 99.8%,), glacial acetic acid (> 99/ 7%) and HPLC grade acetonitrile (> 99.8%) were obtained from Sigma Aldrich, Dorset, UK. Chlorhexidine digluconate (CHG, 20% w/v in water) and sodium octane-1-sulfonate monohydrate (99 + % crystalline) were obtained from Alfa Aesar, Lancashire,UK. Ethanol absolute was obtained from VWR International Ltd. (Leicestershire, UK). Glycerol was purchased from Acros Organics. Franz-type diffusion cells were obtained from Soham Scientific (Soahm, Cambridgeshire, UK). The Dermapen® device was obtained from Dermapen Ltd., Knaresborough, UK. The Zeta Profilometer optical microscope was obtained from KLA-Tenor, Milpitas, CA, USA. The Aquaflux™ transepidermal water loss (TEWL) meter (model AF200) was obtained from Biox Systems Ltd., London, UK. Parafilm® was obtained from Sigma Aldrich, Dorset, UK. Shimadzu Prominence HPLC system with an SPD M20 diode array detector was obtained from Wolverton, UK. A Thermo Scientific guard column with replaceable guard cartridges (C_18_ 10 mm, 5 μm) and a reverse-phase HPLC column (C_18_; dimension, 150 × 4.6 mm, 5 µM) were obtained from Fischer Scientific, Loughborough, UK. Syringe filters (0.2 μm, 15 mm diameter), HPLC vials (1.5 mL crimp neck vial, 32 × 11.6 mm) and crimper caps (1.0 mm), D-Squame™ tape strips standard) were obtained from Cuderm corporation, USA. Loctite® Super Glue adhesive and Sellotape™ were obtained from Lyreco (Shropshire, UK). The Time-of-Flight Secondary Ion Mass Spectrometry (ToF–SIMS) IV instrument was obtained from IONTOF, GmbH, Münster, Germany.

### *In Vitro* Skin Simulant Insertion

An *in vitro* skin simulant insertion study was employed to elucidate the penetration profile (depth and pore size) of microneedle penetration as a function of length and array size. The insertion experiment is based on a previously published work by Larrañeta *et al*. (37). In brief, a polymeric film (Parafilm M®)) was utilised as a skin simulant. Eight layers of Parafilm M® were stacked onto each other on a cork mat that mimics underlying muscles. The Dermapen® was applied to the Parafilm M® stack for 10 s. Following removal of the Dermapen® from the PF layers, each layer was separated, labelled and viewed under a light microscope. Table I shows the different microneedle treatments applied to the Parafilm M® stack.

**Table I:** Skin treatments for determining the effect of microneedle length and array on penetration. n = 3

Treatment	Array size
Dermapen® 750 µm	12
Dermapen® 750 µm	36
Dermapen® 1000 µm	12
Dermapen® 1000 µm	36

### Dye Binding Study

In order to determine the effect of microneedle length and array size on the penetration profile of the Dermapen® into *ex vivo* skin, a dye binding study was conducted. The skin insertion experiment was conducted as previously described (28). *Ex vivo* porcine skin was used for the insertion study due to histological similarity to human skin (38). Prior to microneedle insertion, the skin was allowed to equilibrate to room temperature for up to 30 min. The skin was placed on a cork mat to mimic the underlying muscle and fat tissue. The skin samples were treated with the Dermapen® for 10 s followed by the application of 200 µL gentian violet post-microneedle insertion. The treatments applied to *ex vivo* porcine skin were similar to that of the *in vitro* skin simulant study as shown in Table I. The dye was left on the skin surface for 50 min to allow it to permeate into the microneedle channels, after which excess dye was removed using a 70% alcohol wipe. Following the dye binding study, the skin samples were cryosectioned and imaged using the Zeta Profilometer optical microscope.

### CHG Loaded 2-Hydroxyethylcellulose (HEC) Gel Preparation

Gels were formulated by firstly mixing all wet ingredients (water, ethanol, CHG, glycerol), followed by the slow addition of HEC into the mixture. The formulations were allowed to set for 24 h at room temperature before the *ex vivo* skin permeation experiment. The following formulation shown in Table II was used in this study based on the optimised formulation previously reported (14).

**Table II:** 2-hydroxyethylcellulose (HE)C gel formulations loaded with CHG

CHG (% w/w)	Clycerol (% w/w)	Ethanol (% w/w)	HEC (% w/w)	Water (% w/w)
1	1	60	0.5	Up to 100%
4	1	60	0.5	Up to 100%

### Skin Permeation of CHG Formulations

CHG skin permeation was evaluated *ex vivo* using a Franz-type diffusion cell. Transepidermal water loss (TEWL) was measured for each skin sample to check its integrity (39). Suitable skin samples were placed on a cork board for support and the Dermapen® applied vertically to the skin at the lowest microneedle oscillation speed (8000 RPM) for 10 s. Post-microneedle application, the skin was immediately placed in the diffusion cell apparatus and the formulations were applied to the skin. The Franz-type cells, with a phosphate buffer saline (pH 7.4) receptor fluid were placed in a water bath set to 37 °C for the duration of the experiment. Samples were collected regularly over a period of 24 h. Thereafter, the cells were disassembled and excess formulation remaining on the skin surface was removed using absorbent paper towel. The skin air dried for one hour, after which 21 consecutive D-squame™ tape strips were firmly pressed onto the treated area of skin using a roller and quickly removed from each skin sample to remove *stratum corneum* corneocytes, with tapes collected as described previously as individual tape strips or pooled samples (14, 21, 40, 41). CHG was extracted from all tape strips into 5 mL of mobile phase for HPLC analysis. Tape stripping studies for ToF–SIMS analysis also used the method described above, however tape strips for ToF–SIMS analysis were placed sticky-side up onto glass microscope slides following application to the skin, and secured in place with double-sided Sellotape™. For cryosectioning studies both vertical and lateral sectioning was utilised in order to elucidate the distribution of the drug within the skin (21).

### HPLC Analysis

HPLC analysis of both CHG receptor phase and tape strip samples used the method detailed previously (42). A Thermo Scientific guard column with replaceable guard cartridges was used to ensure HPLC pressure remained stable throughout the study. The limit of detection (LoD) and limit of quantification (LoQ) were calculated from this calibration graph according to the following Eqs.  (43):

1 $$LoD=\frac{3\times standard\;deivation}{slope}$$

2 $$LoQ=\frac{10\times standard\;deivation}{slope}$$

The mobile phase was used as the solvent for all CHG extractions and was validated on the Shimadzu system (r^2^ value 0.9992). The LoD was calculated to be 0.362 µg/mL and the LoQ was calculated to be 1.098 µg/mL.

### ToF–SIMS Analysis

The ToF–SIMS methods used in this study have been demonstrated in previous studies (23, 44, 45) and was performed using a ToF–SIMS IV instrument with a Bi_3_^+^ cluster source. A primary ion energy of 25 keV was used; the primary ion dose was preserved below 1 × 10^12^ per cm^2^ to ensure static conditions. A pulsed target current of approximately 0.3 pA and post-acceleration energy of 10 keV were employed throughout the sample analysis. The mass resolution for the instrument was 7000 at *m/z* 28. The scanned area of the tape strips samples was 4 mm × 4 mm, encompassing the skin area exposed to the formulation during the diffusion cell experiments. The raster size for individual tiles for the 4 mm × 4 mm tape strip analysis was 500 × 500 µm. The analysis was carried out at 1 shot/pixel with a total of one scan. An analysis area of 1.5 mm × 3 mm was employed for the skin cross-sections. The raster size for individual tiles for the 1.5 mm × 3 mm skin cross-section analysis was 400 × 400 µm. The analysis was carried out at 1 shot/pixel with a total of three scans. Both sample types were analysed at a pixel density of 100 pixels/mm. An ion representing biological material and therefore indicative of skin (skin marker) was identified as CN^−^ and was used to threshold the data sets from tape strips by using the secondary ion images to create a region of interest, in this instance the area that is only covered by the corneocytes. The images are processed so that any region that falls below the signal of the ions originating from the corneocytes are removed. This then enables exclusion of ions originating from the adhesive tape material found between the furrows in the stripped corneocytes and hence the data can be selectively analysed from the corneocyte material only. CN^−^ is a common fragment observed in organic materials such as biological specimens. Therefore, this secondary ion was used to track the presence of corneocytes extracted on the tape strips. The data was reconstructed to remove the data from the adhesive tape material found between the fissures in the stripped skin and therefore the data was only analysed from the skin material.

### Statistical Analysis of Data

Statistical analysis was conducted using GraphPad Prism 7.02 software. Data are described statistically and shown as previously reported (14). Data are shown as mean ± standard error of mean (SEM). When comparing two groups an unpaired t-test analysis was used, while one-way analysis of variance (ANOVA) with Tukey’s multiple comparisons tests was used to compare multiple groups. P values < 0.05 were considered statistically significant.

## RESULTS AND DISCUSSION

### Microneedle Insertion Study

**Fig. 1:**
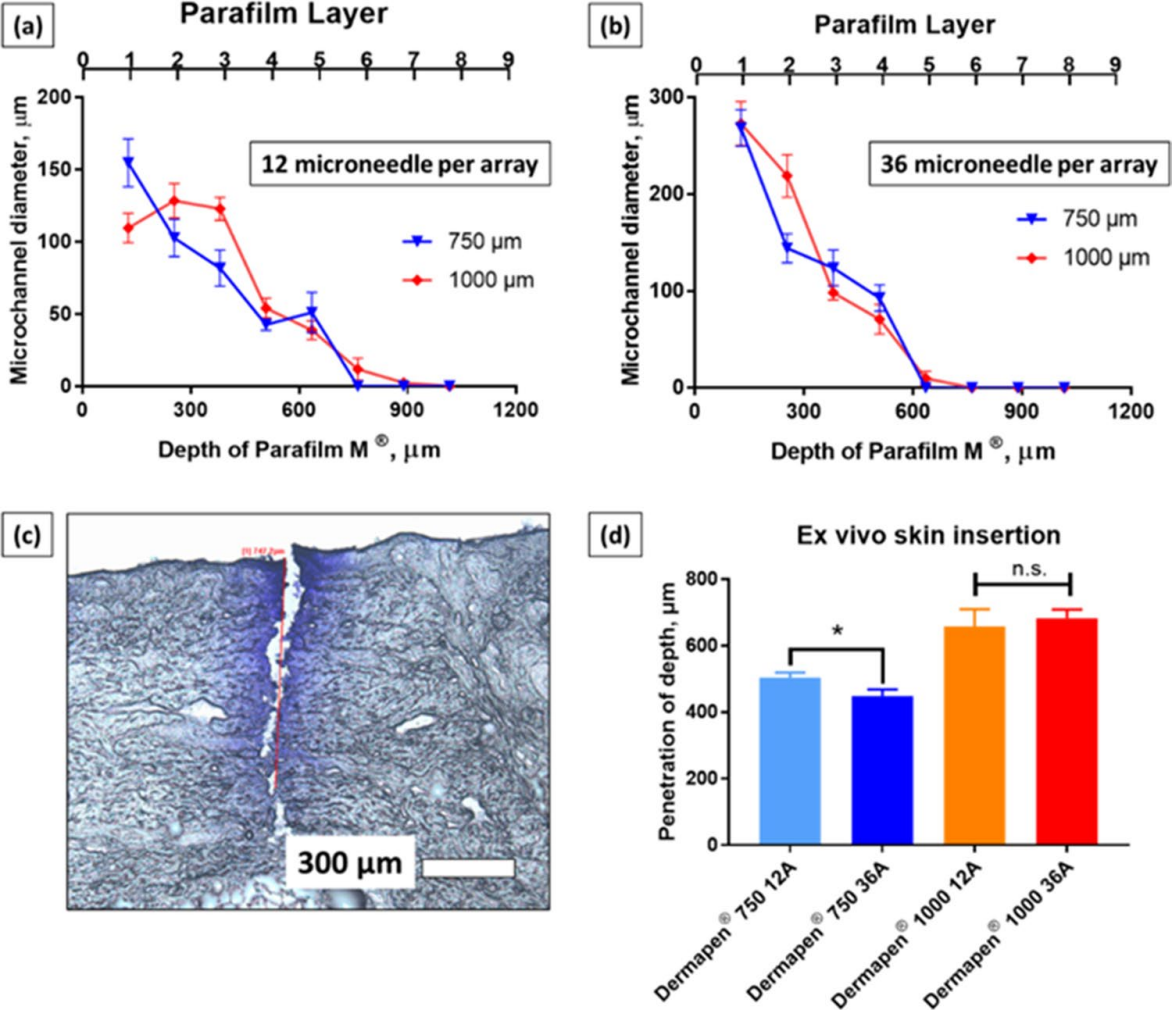
Insertion profile of Dermapen microneedle into Parafilm® layers when equipped with (**a**) 12 Array micronnedle cartridge (**b**) 36 Array micronnedle cartridge. Data are expressed as mean ± SEM for n = 10. (**b**) An example of a microchannels created in *ex vivo* porcine skin visualised via Gentian violet dye binding study (**d**) Microneedle penetration depth for Dermapen® of different microneedle length (750 µm and 1000 µm) and array size (12 Array and 36 Array) into *ex vivo* porcine flank skin. Data are expressed as mean ± SEM for n = 10. Differences were calculated using one-way ANOVA, followed by Tukey’s post hoc test, and deemed significant at p < 0.05. n.s = not statistically significant at p > 0.05

A general trend of increasing microneedle insertion depth with increasing microneedle length was observed, along with a decrease in microchannel diameters with increasing Parafilm® layers as shown in Fig. 1a-b.

The results in Fig. 1a-b indicate that the diameter of the microneedle channels were wider from the 36 Array microneedle than the 12 Array microneedle array on the first layer of Parafilm®, after which the size of the microchannels appeared to be similar irrespective of array size. It can also be seen from Fig. 1a-b that the error bars were relatively small, indicating that the diameter of the microchannels were relatively consistent and similar. The formation of consistent microchannels especially in the first Parafilm® layer is significant as this layer notionally represents the *stratum corneum*, which is the main barrier for the delivery of hydrophilic active agents into the skin. Figure 1c shows the successful penetration of the Dermapen® microneedles into *ex vivo* porcine skin, indicating that the region surrounding the microneedle pores retained a normal structure consistent with an intact *stratum corneum*. However, the microneedle channels displayed a deep indentation with disrupted *stratum corneum*. The mean penetration depth for Dermapen® 750 µm 12 Array and Dermapen® 750 µm 36 Array were 503 µm and 482 µm, respectively (Fig. 1d), a statistically significant reduction for the same microneedle length with an increase in the number of microneedles per array (46). In contrast, when the microneedle length was increased to 1000 µm, the microneedle penetration depth was similar and may be attributed to the decrease in collagen and elastin levels with increasing skin depth (47).

Figure 1 indicates that all the microneedle lengths evaluated resulted in insertion depths that were greater than 400 µm, suggesting that all the lengths evaluated would reach this target depth where subdermal bacteria reside. Guided by this data, the 750 µm length microneedles (Dermapen®) was selected for the skin permeation study as this would allow the microneedles to generate sufficiently deep microchannels for the delivery of CHG to treat subepidermal bacteria. Microneedles with lengths of 1000 µm were deemed too long and would result in increased pain among patients (48).

### Permeation of CHG Formulations Skin Permeation Studies

#### Depth of CHG Permeation Evaluated by the HPLC Analysis of Tape Strips

**Fig. 2:**
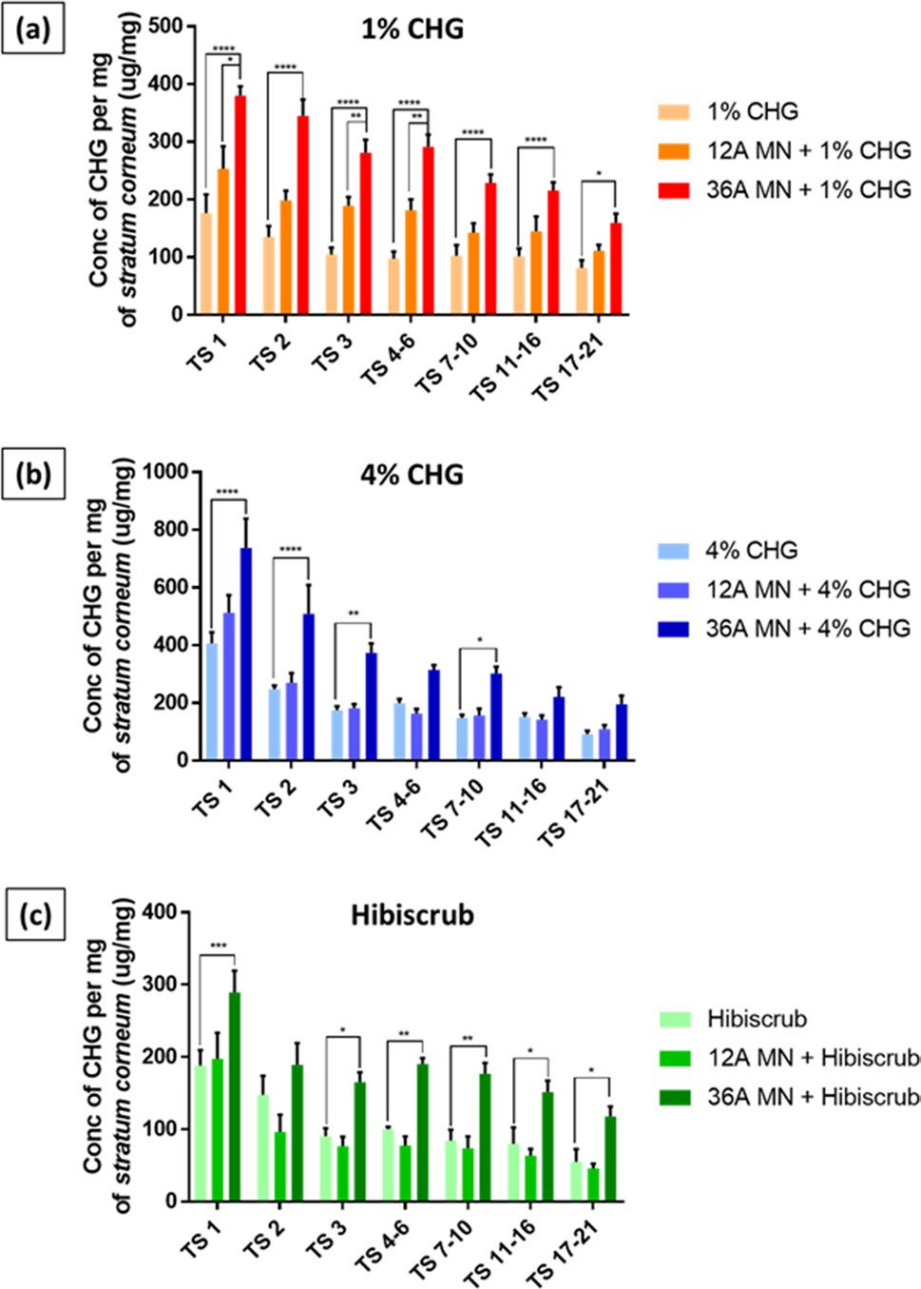
Results of tape stripping studies following Franz-type *in-vitro* diffusion cell experiments of CHG per mg of stratum corneum (SC) material weighed gravimetrically: (**a**) concentration of CHG detected from formulations (all containing 1% w/v CHG) that were delivered with and without the assistance of stainless steel microneedle; (**b**) concentration of CHG detected from formulations (all containing 4% w/v CHG) that were delivered with and without the assistance of stainless steel microneedles; (**c**) concentration of CHG detected from the commercial benchmark formulation, Hibiscrub® (containing 4% w/v CHG) that were delivered with and without the assistance of stainless steel microneedles. Data are expressed as mean ± SEM for n = 4 analytical repeats. MN-refers to microneedles, Differences were calculated using one-way ANOVA followed by Tukey’s post hoc test and deemed significant at p < 0.05

Following diffusion cell experiments the amount of CHG extracted from respective tape strips is shown in Fig. 2. In general, it can be seen that permeation profile of CHG into the *stratum corneum* was much higher when the skin was pre-treated with Dermapen® prior to topical application of respective formulations than with just the formulation alone. The exception to this is for the Hibiscrub® study, compared to the 12-array pre-treatment followed by Hibiscrub®, where there is no difference in the permeation observed from these two formulations. When comparing the two different microneedle arrays (36-array vs 12-array) used, it can be seen that the 36-array enhanced the permeation significantly more (*p* < 0.05) than the 12-array system for all formulations (Fig. 2). Overall, the microneedles gave the hypothesised increase in skin permeation and the results in Fig. 2 suggest that this outcome is extended with repeated use of the Dermapen® device prior to the topical application of CHG-containing formulations.

The data in Fig. 2 indicates that solid microneedle pre-treatment enhanced the permeation profile of CHG significantly into the *stratum corneum* from the HEC gels, relative to Hibiscrub®. The application of Hibiscrub®, in which CHG is formulated in a solution of water and isopropyl alcohol, to skin that has been pre-treated with solid microneedles results in an increase in permeation of the CHG solution via the aqueous microneedle channels, leading to enhanced CHG permeation relative to intact skin in all but one case (Hibiscrub® vs. 12-array microneedle pre-treatment Hibiscrub®). However, the duration that the microneedle channels remained open would be a pivotal factor in determining the extent of CHG permeation into the *stratum corneum* layers (49, 50). The tape strip data also indicated that the 36-array was more effective than the 12-array microneedle in delivering an increased amount of CHG into the skin after 24 h, a finding consistent with other studies (51).

HEC was utilised as a gelling agent due to its well established safety profile and capability to form a film on the skin surface (52). The application of HEC gels on the skin resulted in the formation of an occlusive film which was not observed when the skin was treated with Hibiscrub®. The formation of this HEC film on the skin surface following topical application may result in an occlusive effect at the site of application, an effect which has been widely reported to prolong the opening of microneedle channels (53). This is potentially responsible for significantly greater permeation of CHG into microneedle pre-treated skin, compared to delivery in skin that had not been pre-treated with microneedles. However, such a trend was not observed when CHG was administered from the Hibiscrub® formulation and where no film was formed on the skin surface.

Figure 2 also indicates that changing the number of microneedle arrays used also provides a simple way to control the amount of drug administered into the skin, with a higher array number resulting in a higher amount of drug administered into the skin.

#### ToF–SIMS Visualisation of CHG Distribution From Tape Strip Studies

**Fig. 3:**
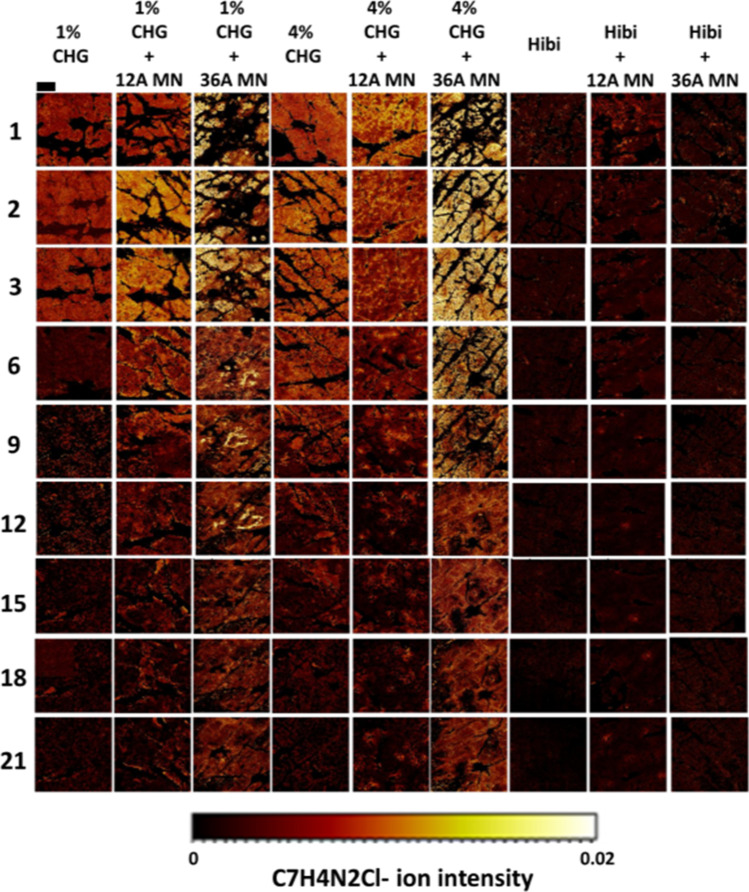
CHG ion C_7_H_4_N_2_Cl- secondary ion distribution maps on tape strip surfaces for tape strips 1, 2, 3, 6, 9, 12, 15, 18, 21 from treatment groups Hibiscrub® 4% w/v solution, 4% w/v CHG gel and 1% CHG gel with and without the assistance microneedle as a skin pre-treatment following Franz-type in-vitro diffusion cell experiments. The secondary ion distribution provides a visual representation to visualise the spatial distribution of CHG following different skin treatment strategies

HPLC analysis of tape strips allowed us to quantify how CHG concentration varied within the *stratum corneum*. It is a method, however, that provides limited information on spatial distribution within individual *stratum corneum* layers. To complement this analysis, permeation of CHG was also assessed by monitoring the fragment ion of the permeant, C_7_H_4_N_2_Cl^−^_._ It can be seen from Fig. 3 that normalised ion intensity for C_7_H_4_N_2_Cl^−^, as a function of tape strip number, follows a trend analogous to that observed in the HPLC analysis of tape strips (Fig. 2). In general, a trend of decreasing CHG ion intensity with increasing tape strip number was observed (Fig. 3).

The results presented in Fig. 3 indicate that there is little difference in CHG permeation on each tape strip, at the same depth (represented by tape strip number) between the 1% w/v CHG and 1% w/v CHG microneedle 12 Array, and again between 4% w/v CHG and 4% w/v CHG 12 Array studies.

As the tape strip images provide information on the distribution of the CHG ion within each tape strip, it can be seen that skin that has been pre-treated with a high-density microneedle array (36 Array) results in enhanced lateral permeation of CHG within the *stratum corneum* relative to the Hibiscrub®. This suggests that pre-treating the skin with a high-density microneedle array may enhance the homogenous distribution of CHG within the deeper layers of the *stratum corneum* which may be of great clinical significance in eradicating pathogenic skin bacteria prior to surgical incision. A similar increase in lateral permeation was not as evident when the microneedle pre-treatment strategy was utilised with Hibiscrub®.

#### ToF–SIMS Analysis of Skin Cross-Sections

With tape stripping of the *stratum corneum* clear information on *stratum corneum* deposition can be derived in a simple and robust manner. Conventionally, liquid chromatography mass spectrometry (LC–MS) is typically employed to quantify drug permeation into skin but is unable to provide a detailed distribution map of drug localisation within the tissue. ToF–SIMS was therefore assessed as an alternative that allowed greater mapping of the drug distribution within skin, and the analysis of skin cross-sections by ToF–SIMS allows us to make a more holistic determination of permeation within the entire skin structure, including the deeper layers beyond the *stratum corneum*. This technique has the further advantage of not requiring any extraction procedure that may complicate or compromise the dermal distribution of CHG within skin tissue (54).

**Fig. 4:**
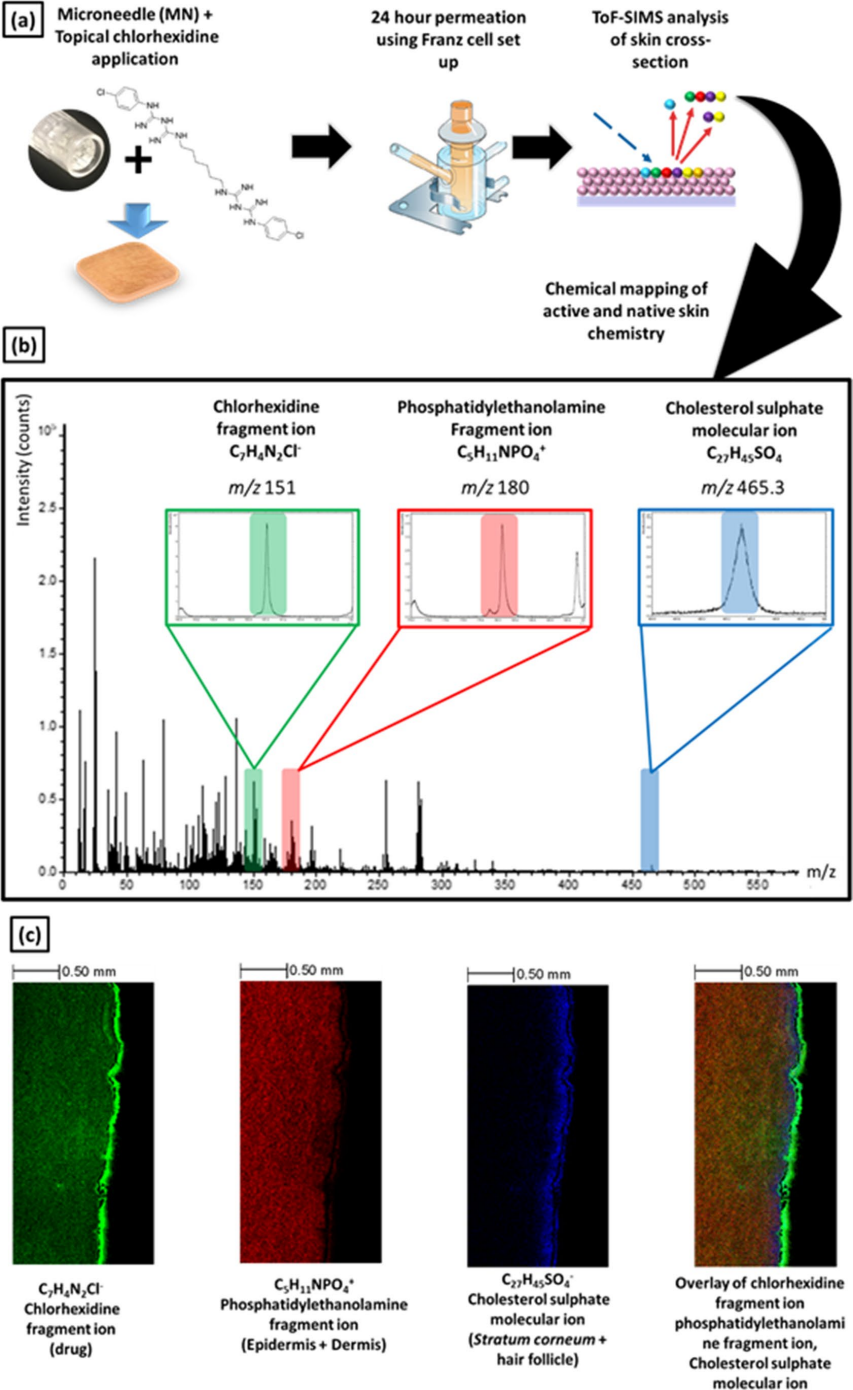
(**a**) Schematic illustrating skin sample analysis following Franz-type diffusion study. (**b**) Negative polarity ToF–SIMS spectra of *ex vivo* porcine skin treated with 4% CHG-HEC gel, where the inset spectrum shows the peak of the chlorhexidine fragment ion at m/z = 151, phosphatidylethanolamine fragment ion at m/z = 180 and cholesterol sulfate molecular ion at m/z = 465.3 (**c**) ToF–SIMS 2D chemical ion maps of phosphatidylethanolamine fragment ion (dermis), chlorhexidine fragment ion (drug) and cholesterol sulfate molecular ion (stratum corneum, epidermis and hair follicle marker) acquired from cross section analysis of *ex vivo* porcine skin tissue after a 24 h permeation experiment. Chemical ion map shows location of skin tissue along with the biodistribution of active and native skin chemistry. The overlay illustrates the potential of ToF–SIMS to detect the localisation of chlorhexidine (active) within the *ex vivo* skin tissue in a label free manner. Abbreviation: CHG, chlorhexidine gluconateScale bar: 500 µm

Following the application of a 4% CHG-HEC gel the treated skin samples were cryosectioned and analysed via ToF–SIMS (Fig. 4a). The ToF–SIMS spectra presented in Fig. 4 (b) highlight three ions of interest; C_7_H_4_N_2_Cl^−^ (a fragment ion of CHG), C_5_H_11_NPO_4_^−^ (the fragment ion of phosphatidylethanolamine, a type of phospholipid native to the skin (20)) and C_27_H_45_SO_4_^−^ (a molecular ion of cholesterol sulphate, also known to be present in skin (55)). The respective secondary ion images (Fig. 4c) demonstrate the spatial distribution of CHG within all the skin layers where C_5_H_11_NPO_4_^−^ was used to map the viable epidermis and dermis (shown in red) while C_27_H_45_SO_4_^−^ was used to detect the presence of the *stratum corneum* and upper epidermis (shown in blue) (27).

**Fig. 5:**
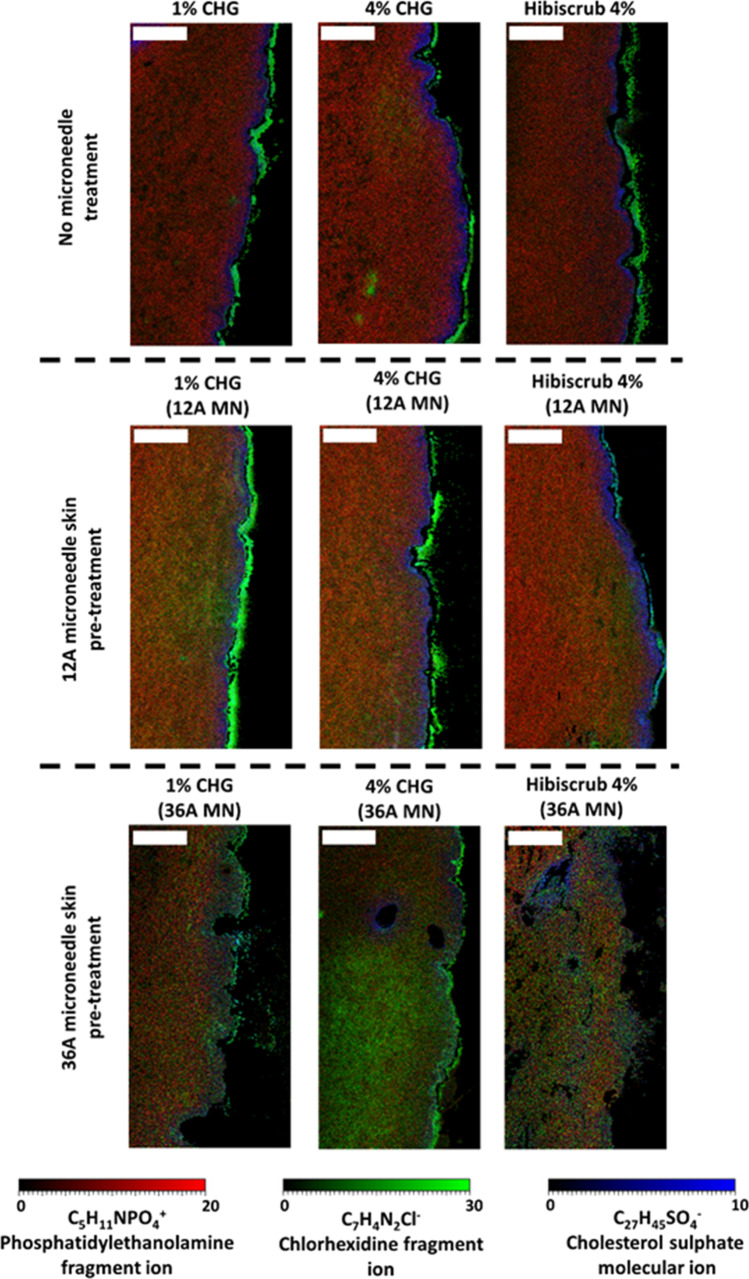
Comparative ion intensity overlain chemical distribution maps from vertical skin cross-sections illustrating the chemical distribution of C_5_H_11_NPO_4_^−^ (phosphatidylethanolamine fragment ion, red), C_7_H_4_N_2_Cl^−^ (chlorhexidine fragment ion, green) and C_27_H_45_SO_4_^_^ (cholesterol sulfate fragment ion,blue) across skin treated with Hibiscrub® 4% w/w, 4% w/w CHG-HEC gel, and 1% w/w CHG-HEC gel across intact and microneedle pre-treated skin. Each image represents an area of 1.5 mm × 3 mm. Abbreviation: CHG, chlorhexidine gluconate, 12A, 12 array microneedle and 36A, 36 array microneedle Scale bar: 500 µm

Figure 5 shows the ToF–SIMS analysis for skin cross-sections from each experiment. These images indicate that CHG ion intensity was largely limited to the *stratum corneum*, with minimal permeation beyond these upper skin layers when intact skin was treated with either 1% w/v, 4% w/v CHG or Hibiscrub®. However, it was apparent then when the skin was pre-treated with the 12 Array microneedle Fig. 4a) a difference in in C_7_H_4_N_2_Cl^−^ ion intensity within the dermis was observed (albeit this is much less pronounced in the Hibiscrub® data). The presence of the C_7_H_4_N_2_Cl^−^ ion within the dermis also became more pronounced when the microneedle density used for skin pre-treatment was increased from 12 to 36 Array. This suggests that increasing the microneedle density used in pre-treating the skin resulted in enhanced delivery and distribution of CHG into the skin, which was more apparent with the 4% CHG sample, which showed substantial proliferation of CHG.

It is apparent from the marker for cholesterol sulfate, C_27_H_45_SO_4_^−^ (indicated in blue) in.

Figure 5 that the *stratum corneum* looks intact when the drug is administered to the skin as either a CHG gel (1% and 4%) or as Hibiscrub®. In contrast, when the drug is delivered to skin that has been pre-treated with a solid microneedle platform, especially the 36-needle Array, disruption in the distribution of cholesterol sulphate ion, C_27_H_45_SO_4_^−^ that suggests that the microneedles have disrupted the *stratum corneum* and facilitate the permeation of CHG into the deeper layers of the skin is observed. The combination of HPLC data, ToF–SIMS analysis of tape strips and skin cross-sections provides a clear illustration of how the concentration of CHG was affected by the use of solid microneedles of different arrays as a pre-treatment prior to formulation application to the skin. The ToF–SIMS analysis enhances this understanding by illustrating drug distribution was affected across the skin when a solid microneedle pre-treatment was used.

It can also be seen from the ToF–SIMS analysis of skin cross-sections that CHG appeared to show a relatively homogeneous distribution within the skin when the formulation was applied on the skin that has been pre-treated with microneedles, relative to intact skin. This may indicate the ability of the drug to diffuse laterally within the water-rich deeper layers of the skin following initial permeation through the microneedle channels. These findings were not reported in previous, similar studies, potentially due to the differences in lipophilicity of the different penetrants in these studies (45, 56).

#### ToF–SIMS Analysis of Lateral Cross-Sections of Skin

**Fig. 6:**
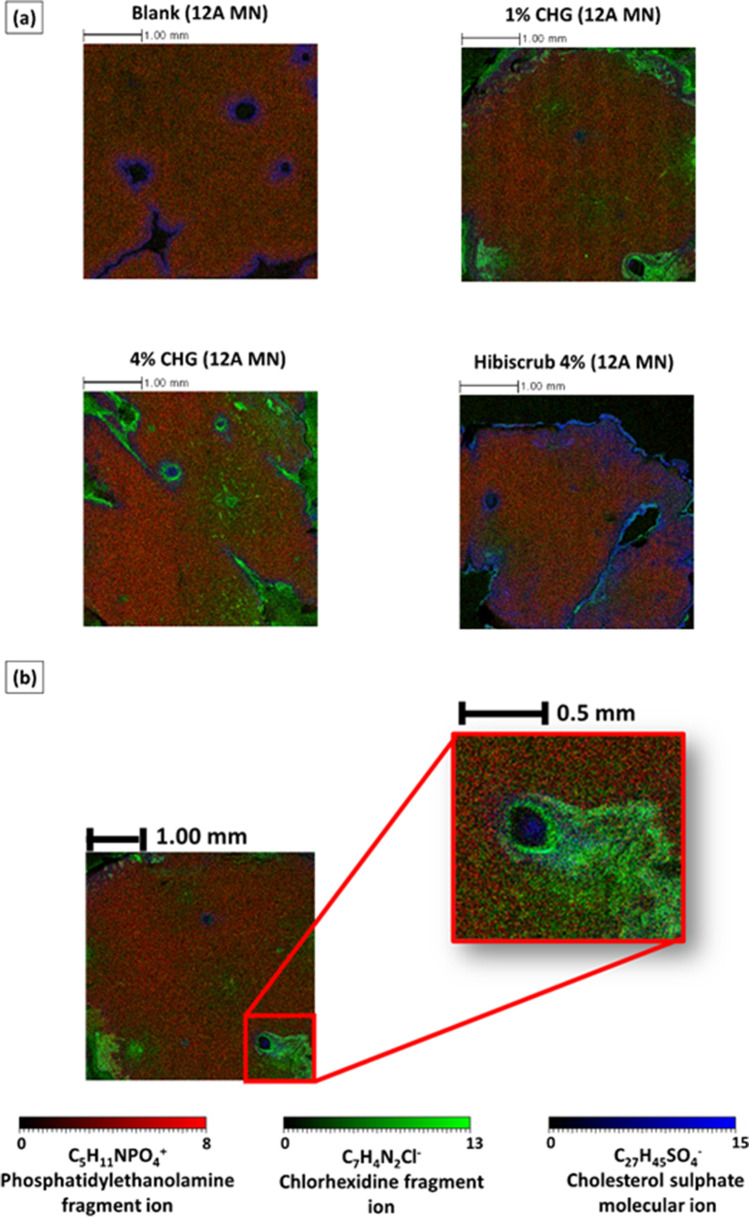
(**a**) Comparative ion intensity overlain chemical distribution maps from lateral skin cross-sections illustrating the chemical distribution of C_5_H_11_NPO_4_^-^ (phosphatidylethanolamine fragment ion, red), C_7_H_4_N_2_Cl^-^ (chlorhexidine fragment ion, green) and C_27_H_45_SO_4_^_^ (cholesterol sulfate fragment ion,blue) across untreated skin and skin treated with Hibiscrub^®^ 4% w/w, 4% w/w CHG-HEC gel, and 1% w/w CHG-HEC gel across microneedle pre-treated skin. Each image represents 4.0 mm × 4.0 mm area of analysis. (**b**) ToF-SIMS image of CHG diffusion within a porcine skin cross section, illustrating higher (C7H4N2Cl^-^) ion intensity in the area immediately surrounding the microneedle channel. Abbreviation: CHG, chlorhexidine gluconate, 12A, 12 array microneedle and 36A, 36 array microneedle Scale bar: 1.0 mm

In Sect. 3.3.2, the lateral distribution of CHG was visualised via analysis of tape strips. However, this approach is limited to the analysis of drug distribution within the *stratum corneum.* ToF–SIMS analysis of lateral skin cryo-sections was conducted in order to examine whether lateral diffusion of CHG is evident in the deeper layers of the skin following microneedle-facilitated delivery. Lateral cross-sections of porcine skin treated with 1% w/v CHG and 4% w/v CHG following a 12 Array solid microneedle pre-treatment step were analysed using ToF–SIMS. In this analysis (Fig. 5), it was apparent that cholesterol sulphate (C_27_H_45_SO_4_^−^) was localised to the upper layers of skin, primarily within the *stratum corneum* (44). However, when the skin was subjected to microneedle insertion, it can be seen that the cholesterol sulphate is now pushed downwards along with the microneedle leading to formation of channels within the dermis with highly localised cholesterol sulphate molecular ion (C_27_H_45_SO_4_^−^) as shown in Fig. 6 (in blue).

It can be seen in Fig. 6a that after a 24-h Franz cell permeation study, porcine skin that was pre-treated with a 12 Array microneedle exhibited lateral diffusion of CHG within the dermal tissues. It can also be seen in Fig. 6b that the CHG ion intensity in the area immediately surrounding the microneedle channels is higher for the skin treated with CHG than for the blank sample that was only pre-treated with the 12 Array microneedle. This suggests that the increase in ion intensity surrounding the microneedle channels was not due the manual insertion of the microneedles but is attributed to the flow of the CHG gels down the channels which subsequently radiate to the vicinal dermal tissues which had not been pierced by microneedles. The visualisation of drug distribution within lateral cross-sections as analysed via ToF–SIMS suggests lateral diffusion within the skin, a concept that was described previously following microneedle application (56). The lateral diffusion of CHG from the microneedle channels into the surrounding water-rich dermal tissue is attributed to physiochemical properties of CHG, i.e. a high aqueous solubility and low log P (8).

Lateral diffusion of small molecules within the skin was initially studied by Johnson *et al.*, where they measured the lateral diffusion coefficients of nine fluorescent probes in human *stratum corneum*-extracted lipids, and found that a low molecular weight was a key determinant in enhancing lateral diffusion (57). Jacobi *et al.* investigated the lateral spreading of 4-methylbenzylidene camphor within the *stratum corneum* and found that it was symmetrical and predominately influenced by the nature of the formulation vehicle (58). These studies were mostly limited to lateral permeation of molecules within the *stratum corneum* and the current work expands further on this concept by providing imaging mass spectrometric evidence of the lateral permeation of drug within deeper skin tissues in a label-free manner. This work is also suggestive of enhanced lateral CHG diffusion with skin pre-treated with microneedles, both within the *stratum corneum* and the underlying dermal tissue, as evidenced by ToF–SIMS images of lateral skin cross-sections.

## CONCLUSION

This study demonstrates the potential of solid microneedles as a skin pre-treatment strategy to enhance the delivery of CHG deeper within the skin. The administration of 1% and 4% CHG-loaded HEC gels on the skin pre-treated with microneedles was found to enhance the delivery of the drug across the *stratum corneum* and into the dermis relative to Hibiscrub®. The permeation enhancement effect was attributed to the generation of aqueous microneedle channels that facilitate the entry of CHG into the skin. In addition, these channels also serve as focal point for CHG to permeate laterally to surrounding dermal tissues, leading to improved antiseptic distribution to the skin prior to surgery. The delivery of CHG onto and into skin that has been pre-treated with solid microneedles demonstrated an improvement in CHG permeation depth and lateral distribution, as evidenced from ToF–SIMS analyses of tape strips and lateral cross-sections. This work suggests that 1% or 4% CHG HEC gel with skin pre-treatment with a Dermapen® device provides a significant enhancement in the localised intradermal delivery of CHG, potentially offering an improved antiseptic effect deeper into the skin than current treatments.

## Supplementary Information

Below is the link to the electronic supplementary material.

Supplementary file1 (DOCX 854 KB)

## Data Availability

The authors are happy to provide further data upon request following a formal request in writing.
