# Supplementary material for: Microneedle-Mediated Permeation Enhancement of Chlorhexidine Digluconate: Mechanistic Insights Through Imaging Mass Spectrometry
Source: Pharm Res. 2022 Jun 10;39(8):1945–58. doi: 10.1007/s11095-022-03309-8 (PMC9314308; doi:10.1007/s11095-022-03309-8)
Supplement: Supplementary file 1 — Supplementary file1 (DOCX 854 KB) [file 11095_2022_3309_MOESM1_ESM.docx]

**SUPPLEMENTARY INFORMATION**

**Graphical Abstract**

**Extended versions of Section 2.4, Section 2.7 and Section 3.1**

**Graphical abstract**


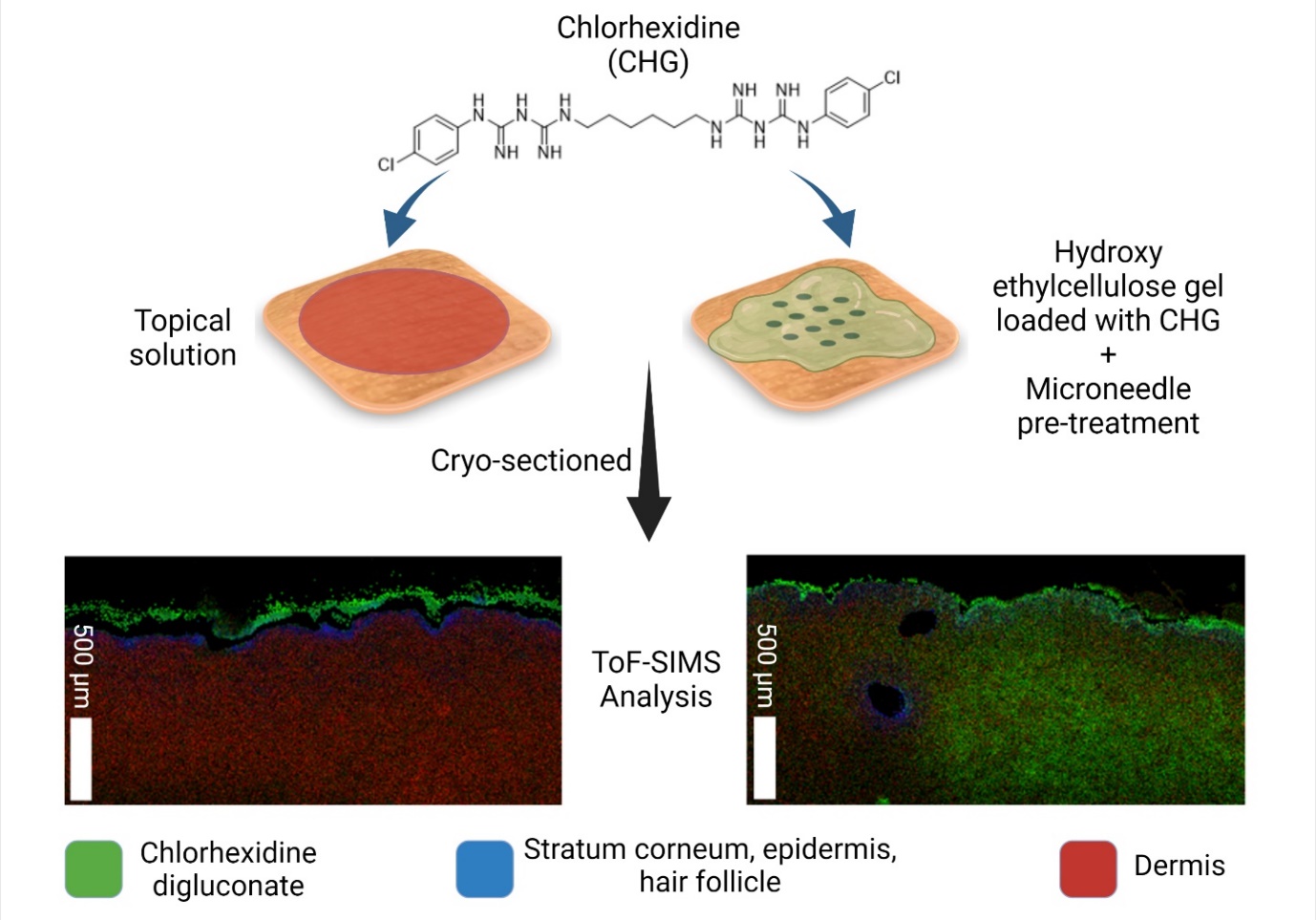


## Section 2.4 Permeation of CHG formulations skin permeation studies

CHG skin permeation was evaluated *ex vivo* using a Franz-type diffusion cell. Transepidermal water loss (TEWL) was measured for each skin sample to check its integrity using a Biox Aquaflux™ meter (model AF200). The TEWL probe was allowed to equilibrate for 2 min before any TEWL measurements of skin samples were taken. Any skin sample with a TEWL measurement 4 × above or below the mean TEWL measurement was discarded ^35^. Skin samples that met the TEWL threshold were placed on a cork board for support and the Dermapen® applied vertically to the skin at the lowest microneedle oscillation speed (8000 RPM) for 10 seconds. Post-microneedle application, the skin was immediately placed in the diffusion cell apparatus and the CHG formulations were applied to the skin. Phosphate buffer saline with (pH 7.4) was used as a receptor fluid for the permeation study. The Franz-type cells were placed in a water bath set to 37^o^C for the duration of the experiment. Samples were taken from the diffusion cell receptor at specific time points over 24hr and subsequently replaced with an equal volume of fresh buffer.

At the end of the 24 h experiment, the diffusion cells were disassembled and excess formulation remaining on the skin surface was removed using absorbent paper towel. Skin was allowed to air dry at ambient temperature for one hour, after which 21 consecutive D-squame™ tape strips were firmly pressed onto the treated area of skin using a roller and quickly removed from each skin sample to remove *stratum corneum* corneocytes ^17^. The first three tape strips were retained separately and the remaining strips were pooled into groups: 4-6, 7-10, 11-16 and 17-21 in order to enable sufficient amount of CHG to be extracted from the latter tape strips for HPLC detection based on our previous work^14^. Tape strips were weighed before and after stripping to allow for gravimetric analysis of tape strip weights and normalisation of CHG content as described previously ^36,37^. CHG was extracted from all tape strips in 5 mL of mobile phase for HPLC analysis. Tape stripping studies for ToF-SIMS analysis also used the method described above, however tape strips for ToF-SIMS analysis were placed sticky-side up onto glass microscope slides following application to the skin, and secured in place with double-sided Sellotape™. With regards to sample cryosectioning both vertical and lateral sectioning was utilised in order to elucidate the distribution of the drug within the skin^17^.

The British Pharmacopoeia method was used initially for the detection of CHG using HPLC, conducted on a Shimadzu Prominence HPLC system with an SPD M20 diode array detector, and the final HPLC method was based upon the method used by Holmes *et al.* (2017)^38^, initially adjusted from Karpanen *et al.* (2008)^5^. The samples were run at a flow rate of 1.5 mL/min at 40 ^o^C through a reverse-phase chromatography column (C18; dimension, 150 × 4.6 mm, 5 µM [Thermo Scientific, United Kingdom]), with UV detection at 254 nm. The isocratic mobile phase consisted of a methanol:water mixture (75:25) with 0.005 M sodium octane-1-sulfonate as an ion pairing reagent (Kudo *et al.* 2002), 0.1% w/v trimethylamine and adjusted to pH 4 with glacial acetic acid. A Thermo Scientific guard column (product code 850-00) with replaceable guard cartridges (Thermo Scientific, C18 10 mm, 5 µm, product code 28105-014001) were used to ensure HPLC pressure remained stable. Cartridges were replaced when the pressure exceeded 20% of the normal pressure limits as recommended by the manufacturer. The HPLC method was validated by analysing a series of standardised CHG concentrations and plotting a graph of peak area versus CHG concentration. The limit of detection (LoD) and limit of quantification (LoQ) were calculated from this calibration graph according to the following equations: LoD (3 × standard deviation)/slope; LoQ (10 × standard deviation)/slope according to the Karpanen *et al.* (2008a) method. The experimental mobile phase was used as the solvent for all CHG extractions and was validated on the Shimadzu system (R^2^ value 0.9992). The LoD was calculated to be 0.362 µg/mL and the LoQ was calculated to be 1.098 µg/mL.

## 2.7 Statistical analysis of data

Statistical analysis was conducted using GraphPad Prism 7.02 or IBM SPSS software. Data are described statistically and shown as previously reported ^5, 14^. Briefly, all data is displayed as the mean + the standard error of the mean (SEM), followed by the sample number, *n*. Data was first assessed for normality and homogeneity of variance using the Shapiro-Wilk and Levenes tests, respectively. If results indicated that the data was parametric then a One-Way ANOVA with Tukey’s post-hoc test was conducted; if results indicated that the data was non-parametric then a Kruskal Wallis ANOVA with Dunn’s post-hoc test was conducted ^14^. When comparing two groups an unpaired t-test analysis was used, while one-way analysis of variance (ANOVA) with Tukey's multiple comparisons tests was used to compare multiple groups. P values < 0.05 were considered statistically significant.

Further, in terms of the ToF-SIMS analysis, it is perhaps important to emphasise that the SIMS area of analysis is substantial and, as such, there are no needs for repeated samples. For example, the method in the manuscript states that an analysis area of 1.5 mm × 3 mm was employed for the skin cross-sections. The raster size for individual tiles for the 1.5 mm x 3 mm skin cross-section analysis was 400 x 400 µm. Thus, although only one tape strip area or cross-sectioned sample area was analysed these macroscopic regions are thought to be more than adequately representative of the overall distribution of CHG in both the X-Y and Z sample coordinates. This is a methodology and an outcome that we have used and published before in other studies, listed below, and these analytical conditions have therefore been applied to our ToF-SIMS methods more broadly.

Meurs J, Scurr DJ, Lourdusamy A, Storer LC, Grundy RG, Alexander MR, Rahman R, Kim DH. Sequential Orbitrap secondary ion mass spectrometry and liquid extraction surface analysis-tandem mass spectrometry-based metabolomics for prediction of brain tumor relapse from sample-limited primary tissue archives. Analytical Chemistry. 2021 Apr 26;93(18):6947-54.

Starr NJ, Khan MH, Edney MK, Trindade GF, Kern S, Pirkl A, Kleine-Boymann M, Elms C, O'Mahony MM, Bell M, Alexander MR. Elucidating the molecular landscape of the stratum corneum. Proceedings of the National Academy of Sciences. 2022 Mar 22;119(12):e2114380119.

McCrorie P, Rowlinson J, Scurr DJ, Marlow M, Rahman R. Detection of Label-Free Drugs within Brain Tissue Using Orbitrap Secondary Ion Mass Spectrometry as a Complement to Neuro-Oncological Drug Delivery. Pharmaceutics. 2022 Mar 5;14(3):571.

Meurs J, Scurr DJ, Lourdusamy A, Storer LC, Grundy RG, Alexander MR, Rahman R, Kim DH. Sequential 3D OrbiSIMS and LESA-MS/MS-based metabolomics for prediction of brain tumor relapse from sample-limited primary tissue archives. bioRxiv. 2021 Jan 1:2020-07.

**Section 3.1. Microneedle Insertion Study**

The *in vitro* assessment of microneedle penetration was performed by applying the Dermapen® onto eight sheets of Parafilm® for 10 seconds at an oscillating speed of 8000 RPM, resulting in the formation of microchannels. Overall, there was a general trend of increasing microneedle insertion depth with increasing microneedle length. On the other hand, there was a decrease in microchannel diameters with increasing Parafilm® layers as shown in Figure 1 **(a-b)**.


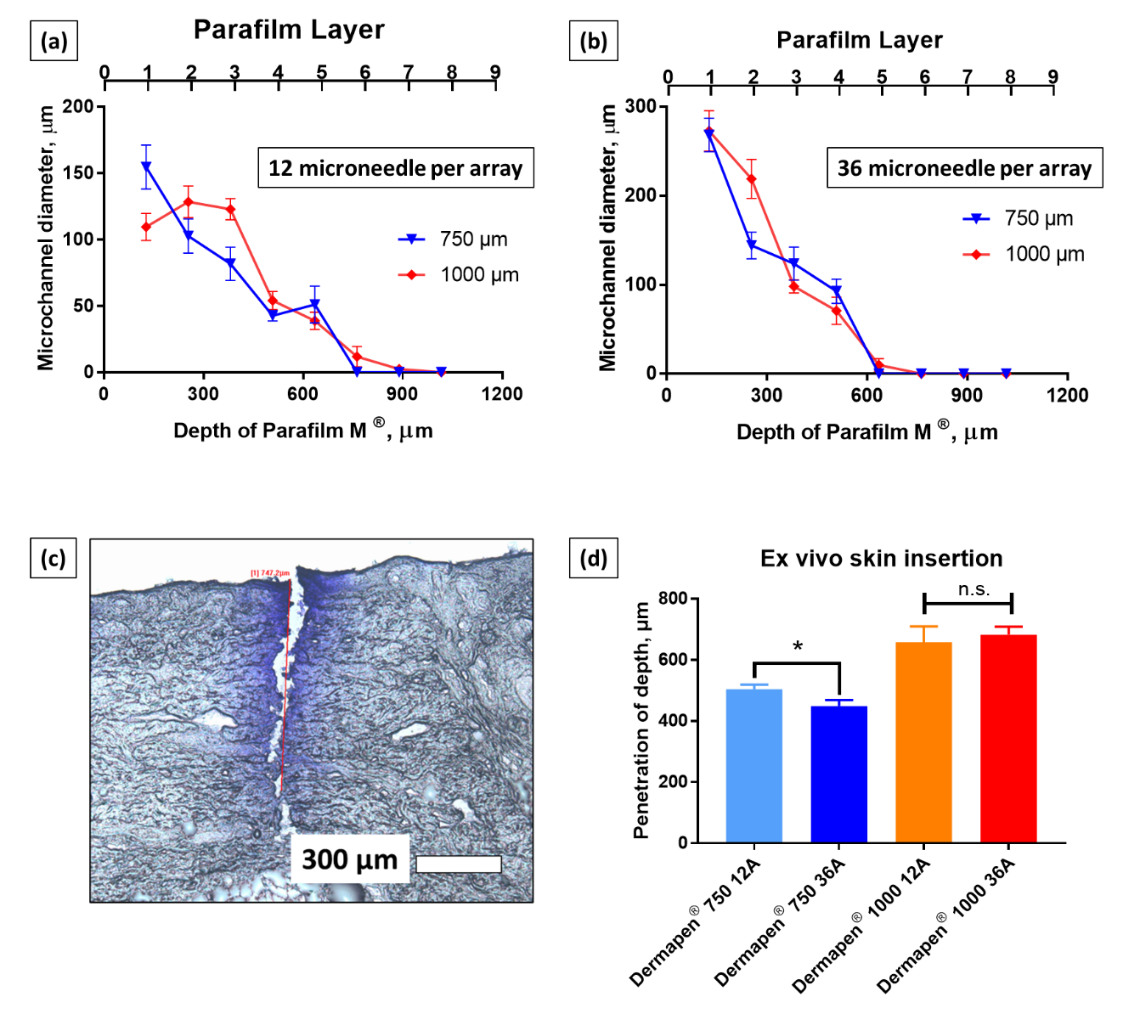


Figure 1 Insertion profile of Dermapen microneedle into Parafilm^®^ layers when equipped with (a) 12 Array micronnedle cartridge (b) 36 Array micronnedle cartridge. Data are expressed as mean± SEM for n=10. (b) An example of a microchannels created in ex vivo porcine skin visualised via Gentian violet dye binding study (d) Microneedle penetration depth for Dermapen® of different microneedle length (750 µm and 1000 µm) and array size (12 Array and 36 Array) into ex vivo porcine flank skin. Data are expressed as mean± SEM for n=10. Differences were calculated using one-way ANOVA, followed by Tukey’s post hoc test, and deemed significant at p<0.05. n.s = not statistically significant at p>0.05

From Figure 1 **(a-b)** it was observed the diameter of the microneedle channels were wider from the 36 Array microneedle than the 12 Array microneedle array on the first layer of Parafilm®, after which the size of the microchannels appeared to be similar irrespective of array size. In addition, it can be seen from Figure 1 **(a-b)** that the error bars were relatively small, indicating that the diameter of the microchannels were relatively similar. Such consistent and homogenous microneedle pore size could be attributed to the oscillating function of the Dermapen® which generates a stamping-like motion on the Parafilm®. This stamping-like motion has been reported to generate a more consistent microneedle insertion force thus overcoming the issues of heterogenous application force across the microneedle array that could result in variation in the pore size and insertion efficiency during skin application ^42^. From the perspective of percutaneous absorption, the formation of consistent microchannels especially during the first Parafilm® layer is very pertinent as this layer typically represent the *stratum corneum*, which is the main barrier for the delivery of hydrophilic active agents such as CHG into the skin. The formation of microchannels in the skin in a reliable and homogenous manner will ensure that any formulation applied to the skin can move through the pores at an equal rate leading to a more homogenous drug distribution across the entirety of the treated skin layer.

In order to evaluate if the insertion profile observed from these *in vitro* characterisations would translate into actual skin tissues, an *ex vivo* skin insertion study was conducted using the Dermapen® device. The microneedle penetration depth was visualised *via* gentian violet staining as shown in Figure 1 **(c)**. The successful penetration of microneedle into the *ex vivo* porcine skin showed that the region surrounding the microneedle pores retained a normal structure consistent with an intact *stratum corneum*. However, the microneedle channels displayed a deep indentation with disrupted *stratum corneum*. The mean penetration depth for Dermapen® 750 µm 12 Array and Dermapen® 750 µm 36 Array were 503 µm and 482 µm respectively as shown in Figure 1 **(d)**. It is apparent that for the same microneedle length an increase in the number of microneedles per array resulted in a statistically significant reduction in the microneedle penetration depth. Such a reduction in microneedle penetration depth with an increase in the number of microneedles per array can be attributed to the “bed of nails effect”. This is a situation where increasing the microneedle density per unit area results in a reduction in the penetration profile of the microneedles as the force applied to the array is now distributed over greater number of microneedles. Ultimately, the pressure exerted at the tip of microneedle will reduce resulting in a lower insertion depth ^43^.

In contrast, when the microneedle length was increased to 1000 µm, the microneedle penetration depth was similar. This suggests that the “bed of nail effect” may be more apparent with shorter microneedle lengths. Such findings may be attributed to the decrease in collagen and elastin levels with increasing skin depth ^44^. When shorter microneedles are applied to the skin, these microneedles will encounter a dense fibrous network of elastin and collagen that provides a significant level of resistance to microneedle insertion. In such instances, an array with lower microneedle number will experience less “bed of nail” effect thus having sufficient pressure to pierce these networks of collagen and elastin resulting in deeper insertion. However, when the Dermapen® was set to 1000 µm, the microneedle penetration profile reached an insertion depth that has a lower density of elastin and collagen networks. At such depth, these network no longer provide sufficient resistance to microneedle insertion thus overriding the “bed of nail” effect.

. It can be seen from Figure 1 that all the microneedle lengths evaluated resulted in insertion depths that were greater than 400 µm, suggesting that all the lengths evaluated would reach this target depth where subdermal bacteria reside. Guided by this skin insertion study data, we selected the 750 µm length microneedles (Dermapen^®^) for the skin permeation study as this would allow the microneedles to generate sufficiently deep microchannels for the delivery of CHG to treat subepidermal bacteria. Microneedles with lengths of 1000 µm were deemed too long and would result in increased pain among patients. This is based on the findings of Gill *et al*. who showed that longer microneedle length resulted in higher pain sensation among healthy human volunteers ^45^.
